# MicroRNAs Associated with Disability Progression and Clinical Activity in Multiple Sclerosis Patients Treated with Glatiramer Acetate

**DOI:** 10.3390/biomedicines11102760

**Published:** 2023-10-12

**Authors:** Ignacio Casanova, María I. Domínguez-Mozo, Laura De Torres, Yolanda Aladro-Benito, Ángel García-Martínez, Patricia Gómez, Sara Abellán, Esther De Antonio, Roberto Álvarez-Lafuente

**Affiliations:** 1 Department of Neurology, Torrejon University Hospital, 28850 Madrid, Spain; i.casanovap@gmail.com (I.C.); lauravdetorres@gmail.com (L.D.T.); gmz.iglesias@gmail.com (P.G.); sabellan@torrejonsalud.com (S.A.); 2 School of Medicine, Universidad Francisco de Vitoria, 28223 Madrid, Spain; 3 Research Group in Environmental Factors of Neurodegenerative Diseases, Instituto de Investigación Sanitaria del Hospital Clínico San Carlos (IdISSC), 28040 Madrid, Spain; garcia.angel23@gmail.com (Á.G.-M.); ralvarezlafuente@yahoo.es (R.Á.-L.); 4 Department of Neurology, Getafe University Hospital, 28905 Madrid, Spain; yolanda.aladro@salud.madrid.org; 5 Department of Radiology, Torrejon University Hospital, 28850 Madrid, Spain; edeantonio@torrejonsalud.com

**Keywords:** multiple sclerosis, glatiramer acetate, microRNAs, no evidence of disease Activity-3

## Abstract

MicroRNAs (miRNAs) are promising biomarkers in multiple sclerosis (MS). This study aims to investigate the association between a preselected list of miRNAs in serum with therapeutic response to Glatiramer Acetate (GA) and with the clinical evolution of a cohort of relapsing–remitting MS (RRMS) patients. We conducted a longitudinal study for 5 years, with cut-off points at 2 and 5 years, including 26 RRMS patients treated with GA for at least 6 months. A total of 6 miRNAs from a previous study (miR-9.5p, miR-126.3p, mir-138.5p, miR-146a.5p, miR-200c.3p, and miR-223.3p) were selected for this analysis. Clinical relapse, MRI activity, confirmed disability progression (CDP), alone or in combination (No Evidence of Disease Activity-3) (NEDA-3), and Expanded Disability Status Scale (EDSS), were studied. After multivariate regression analysis, miR-9.5p was associated with EDSS progression at 2 years (β = 0.23; 95% CI: 0.04–0.46; *p* = 0.047). Besides this, mean miR-138.5p values were lower in those patients with NEDA-3 at 2 years (*p* = 0.033), and miR-146a.5p and miR-126.3p were higher in patients with CDP progression at 2 years (*p* = 0.044 and *p* = 0.05 respectively. These results reinforce the use of microRNAs as potential biomarkers in multiple sclerosis. We will need more studies to corroborate these data and to better understand the role of microRNAs in the pathophysiology of this disease.

## 1. Introduction

There is a great interest and a growing need to develop biomarkers in multiple sclerosis (MS), to better predict the clinical evolution of the disease, the therapeutic response to the different disease-modifying treatments, and to better understand the mechanisms of progression. MicroRNAs are small, highly conserved non-coding RNA molecules, between 20 and 25 nucleotides. They participate in RNA silencing and post-transcriptional modification of gene expression [1], and regulate a multitude of cellular processes [2,3,4]. Moreover, they can be easily, repeatedly, and non-invasively measured in different samples. In recent years different patterns of miRNA expression have been shown in MS patients compared to healthy subjects [5,6,7], relapses versus remission [8], clinical phenotypes [9,10], and radiological patterns [11]. However, there is less information about its use as biomarkers of therapeutic response and as a prognostic predictor of the clinical evolution of the disease [12,13,14]. 

We previously conducted a cross-sectional investigation to correlate the miRNAs profile expression with the Expanded Disability Status Scale (EDSS), cognitive function, and brain volume status in a cohort of MS treated with glatiramer acetate (GA) [15]. This is an immunomodulatory treatment, which consists of a mix of oligopeptides of four amino acids that resemble the myelin basic protein (MBP). 

In this study, we aimed to continue that research and analyze the correlation between the statistically associated miRNAs found in our previous work with the clinical evolution in the follow-up of these patients.

## 2. Materials and Methods

### 2.1. Study Design

We conducted a longitudinal study during a follow-up term of 5 years, with cut-off points at 2 and 5 years. 

Inclusion criteria: relapsing–remitting multiple sclerosis (RRMS) patients treated with GA for at least 6 months attending the MS unit of Torrejón University Hospital and Getafe University Hospital in Madrid. 

Participants were reviewed clinically every 6 months as routine clinical practice, and every time that there was a relapse or any other clinically relevant situation. MRIs were performed every year as routine clinical practice. Those patients that reached the criteria of GA failure were changed to other disease-modifying treatments, as physician criteria, but continued in the study. 

Confirmed disability progression (CDP) was defined as a 6-month confirmed EDSS increase of ≥1.5 points if basal EDSS was 0; a 6-month confirmed EDSS increase of ≥1 point if basal EDSS was between 1 and 5.0; and a 6-month confirmed EDSS increase of ≥0.5 points if basal EDSS was ≥5.5. 

Magnetic resonance imaging (MRI) of the brain was performed one month before the beginning of glatiramer acetate treatment and every year since treatment initiation in 1.5T scanners. The sequences collected for this study were: axial proton density T2-weighted imaging, axial fluid-attenuated inversion recovery (FLAIR) T2, axial T2-weighted imaging, and T1-weighted imaging with gadolinium (Gd) enhancement. To cover the entire brain with contiguous axial sections, a slice thickness of 5 mm was performed. MRI activity was defined as ≥1 gadolinum-enhancing lesion and/or ≥2 new or enlarging T2 lesions. 

No Evidence of Disease Activity-3 (NEDA-3) was defined as not having CDP, MRI activity, or any relapse. 

### 2.2. MicroRNAs Selection and Analysis

Peripheral blood samples were collected from each enrolled patient in redtop vacutainer tubes without additives (BD Vacutainer^®^, Franklin Lakes, NJ, USA), centrifuged at 920× *g* for 15 min at room temperature to separate serum and stored at −80 °C until RNA extraction. Before using the frozen serum for nucleic acid purification, we thaw it at room temperature. To remove cryoprecipitates, we centrifuge 300 μL thawed serum samples for 5 min at 3000× *g* and 4 °C, and we transfer 200 μL of supernatant to a new tube. Cell-free total RNA was extracted using the miRNeasy Serum/PlasmaAdvanced Kit (Qiagen, Hilden, Germany) according to the manufacturer’s protocol. During the RNA extraction process, the UniSp2, UniSp4, and UniSp5 RNA Spike-in mix (RNA Spike-in Kit for RT, Qiagen^®^, Germantown, MD, USA) was added to have a control for the quality of the RNA isolation. The total RNA was reverse transcribed using miRCURY LNA RNA kit (Qiagen, Hilden, Germany) following the manufacturer’s instructions that generate universal cDNA templates for all miRNAs present in the sample. The synthetic UniSp6 RNA spike-in (Qiagen, Hilden, Germany) was added to each sample during this process to have a control for the quality of the cDNA synthesis; and the reaction was performed in the Veriti™ thermal cycle (Applied Biosystems™, Waltham, MA, USA). Prepared complementary DNAs were stored at −20 °C until use. We performed the miRCURY LNA miRNA QC PCR Panel (Qiagen, Hilden, Germany) to analyze the robustness of the RNA isolation process and the quality of isolated miRNA. The panel contains matching locked nucleic acid (LNA) PCR assays for detection of: the RNA Spike-In mix (UniSp2, UniSp4, and UniSp5); the spike-ins UniSp6 andcel-miR-39-3p (not added in our experiments) to monitor cDNA synthesis; theUniSp3 IPC (inter-plate calibrator) to check if the qPCR was successful; four potential endogenous: miR-103-3p, miR-191-5p, miR-30c-5p, and miR-124-3p; andmiR-451a and miR-23a-3p that serve as hemolysis marker. MiRNA-specific quantification was performed using miRCURY LNA SYBR Green kit (Qiagen, Hilden, Germany) according to the manufacturer’s instructions, in a LightCycler 96 instrument (Roche Applied Science, Basel, Switzerland). We performed the miRCURY LNA miRNA Custom PCR Panels using only the samples with successful results in the miRCURY LNA miRNA QC PCR Panel. A total of 6 miRNAs statistically associated with clinical disability and brain atrophy in a previous work [15] were included in the miRCURY LNA miRNA Custom PCR Panels: miR146a.5p, miR-9.5p, miR-126.3p, miR-200c.3p, miR-138.5p, and miR-223.3p (Table 1), apart from the four potential endogenous, the spike-ins UniSp6; and the UniSp3. MiRNA-specific quantification was performed using miRCURY LNA SYBR Green kit (Qiagen, Hilden, Germany) according to the manufacturer’s instructions, in a LightCycler 96 instrument (Roche Applied Science). All reactions were run as duplicates. Normalization was performed using the mean expression of two endogenous miRNAs: miR191-5p and miR30c-5p. The normalized cycle quantification (Cq) value was calculated as mean Cq—endogenous Cq.

### 2.3. Statistics

Statistical Package for Social Sciences, version 19.0 (IBM SPSS, Inc., Chicago, IL, USA) was used for statistical analysis. We described numerical variables expressed as with median and interquartile range and categorical variables as percentages. The correlation between miRNAs and EDSS progression was determined using backstep multivariate regression. Only the 6 microRNAs were included in the multivariate regression. Other variables were not included due to the small and homogeneous group. The tolerance limit was established at 0.01. The statistical significance for variable exclusion (POUT) for the sequential analysis was defined at *p* > 0.10. Association between miRNAs and clinical outcomes (clinical relapse, MRI activity, and/or CDP), alone or in combination (NEDA-3) was studied with a non-parametric test (U Mann–Whitney). Statistical significance was set at *p* ≤ 0.05. 

## 3. Results

We included 26 patients. The sample was composed of a typical early RRMS population, with female predominance, young age, and mild disability (Table 2). There was no statistically significant difference in the distribution of any miRNA regarding sex or age.

NEDA-3 at 2 and 5 years was achieved by 70.8% and 56.5% of the patients, respectively. At the end of the study, only 20.81% of the patients remained on GA, while 42.3% were classified as therapeutic failure, and 30.8% had changed due to other reasons (side effects or patient preferences). There was one loss of follow-up (Table 3).

**Figure 1:**
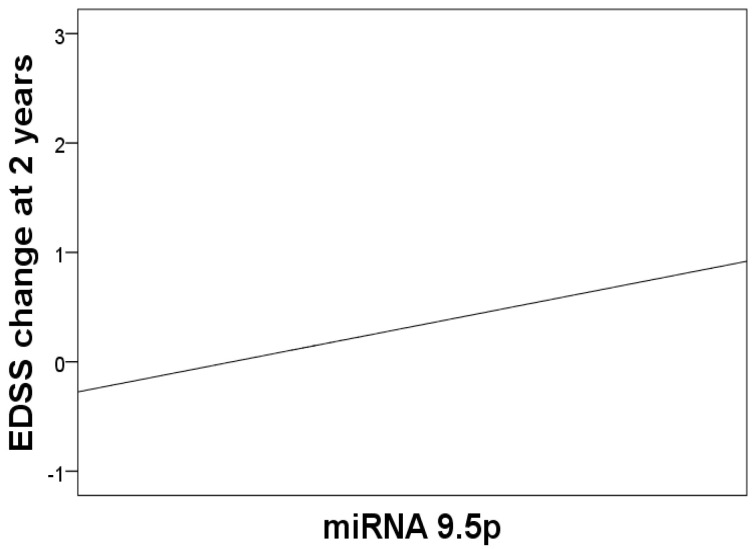
Association between miR-9.5p and EDSS progression at 2 years (backstep multivariate regression analysis).

We found an association between mir-9.5p and EDSS progression at 2 years with multivariate regression analysis (β = 0.23; 95% CI: 0.04–0.46; *p* = 0.047) (Figure 1). We did not find any miRNA associated with EDSS progression at 5 years (Table 4). 

**Figure 2:**
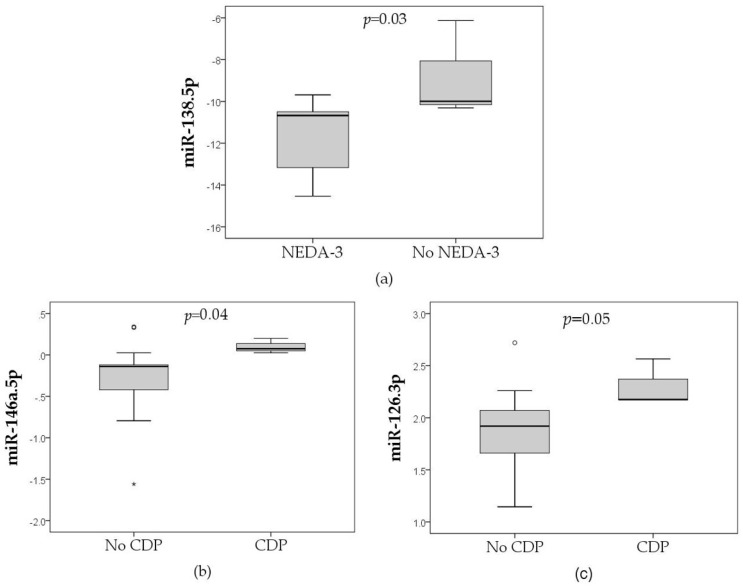
Expression levels of miR-138.5p, miR-146a.5p, and miR-126.3p depending on different clinical variables. (**a**) Association between expression levels of miR-138.5p and NEDA-3 (no CDP, magnetic resonance imaging activity or any relapse) at 2 years, n = 12. (**b**) Association between expression levels of miR-146.5p and confirmed disability progression (CDP), n = 20. (**c**) Association between expression levels of miR-126.3p and CDP, n = 20.

With non-parametric analysis, we found an association between miR-138.5p and NEDA-3 at 2 years (lower values in NEDA-3 patients; *p* = 0.033). We also found an association between higher values of miR-146a.5p and miR-126.3p and CDP progression at 2 years (*p* = 0.044 and *p* = 0.05, respectively) (Figure 1). We did not find any miRNA associated with relapse and/or MRI activity at 2 years and with any clinical outcome at 5 years of evolution (Figure 2, Table S1). 

## 4. Discussion

In this study, we have corroborated some findings of a previous work [15]. From the 6 miRNAs correlated previously with several measurements of MS, we have found an association again with 4 of them (miR-138.5p, miR-126.3p, miR-9.5p, and miR-146a.5p) (Table 5). These data strengthen the value of these miRNAs as biomarkers in MS patients treated with GA. Moreover, all these associations were established with different outcomes of MS progression (EDSS, CDP, and/or NEDA-3) and not with MS activity (either relapse or new MRI lesions). We hypothesize that this selective association with progression could be explained by the methodology used to preselect the miRNA candidates (Anaxomics^®^, Barcelona, Spain), which was centered on MS pathology and cognitive dysfunction. These aspects would be more related to degeneration than to inflammation. These results would highlight the importance of these miRNAs in the pathogenic mechanism of neurodegeneration in this disease. 

Robust evidence exists with mir-9.5p and miR-146.5p. In our previous work, miR-146.5p was positively and negatively associated with EDSS and Symbol Digit Modalities Test (SDMT), respectively. In this study, we also found higher values of these miRNAs in patients with CDP. MiR-146.5p has been consistently found upregulated in MS, both in different samples (cerebrospinal fluid, blood, active MS plaques) [16,17,18], as well as in different populations [19,20]. In addition to this, elevated levels of miR-146.5p have been correlated with disability progression and unfavorable prognosis [18], as in our two studies. MiR-146.5p exerts anti-inflammatory effects on the innate immune system (promotes M2 and inhibits M1 macrophages reactions, reduces inflammatory cytokines) and facilitates the differentiation of oligodendrocytes precursor cells (OPC) by inhibiting toll-like receptor-2 (TLR2) and Interleukin 1 receptor-associated kinase 1 (IRAK1) [16,21]. It has been demonstrated that this upregulation is due to a negative feedback loop trying to counterbalance the higher inflammatory state [22,23,24]. In fact, their values are decreased after different disease-modifying treatments [18,25]. Treatment with miR-146.5p mimics reduced the severity of an Experimental Allergic Encephalomyelitis (EAE) model and enhanced its remyelination [26]. 

MiRNA-9.5p is a proinflammatory molecule that could play a pathogenic role in MS through several mechanisms, such as facilitating M1 macrophage reaction [27], increasing Th17 differentiation [28,29], and promoting microglial activation [30]. Higher values have also been described in EAE models of the disease [31,32]. In our study, this microRNA was associated with EDSS progression at 2 years after multivariate regression, which is in the same direction as the association found with EDSS and thalamus atrophy in our previous work. For these reasons, we think that microRNA-146.5p and microRNA-9.5p, and their target genes, could be potential therapeutic agents to modulate the clinical course in MS.

MicroRNA 126.3p is located in endothelial cells and participates mainly in angiogenesis and cell migration [33,34]. There have also been demonstrated some functions in the immune system, such as chemokine production or suppression of Th2 [35]. Moreover, it has been associated with natalizumab pharmacodynamics and risk of progressive multifocal leukoencephalopathy [36]. Its effects on MS pathology are unknown and with mixed results. Most of the studies describe lower levels of miR-126 in MS, as well as in other autoimmune diseases, linking these lower values to a higher inflammatory state [36,37]. However, other works find opposite results, with higher values of miR-126 in RRMS lymphocytes [38], augmentation of this molecule during relapses, and a reduction with natalizumab treatment [39]. We understand that these discrepancies between studies might be explained by differences in their methodology. With these results, we could not propose a mechanism of action of miR-126.3p. However, the negative association with SDMT and the progression of the disease in our two projects reinforce the possible utility of this microRNA as a biomarker of a worse prognosis.

MicroRNA 138.5p is a potent tumor suppressor that targets many different genes related to apoptosis, proliferation, invasion, and migration [40,41,42]. It has been linked to several cancers, but it also has some effects on the central nervous system, albeit with opposing mechanisms. On the one hand, it has been associated with a reduction of neuroinflammation through the downregulation of caspase I [43] and with an increase in oligodendrocyte differentiation [44]. On the other hand, it has been related to cognitive impairment due to an overexpression of neurodegenerative and a reduction of neuroprotective proteins [45,46,47] and as a negative regulator of dendritic spine morphogenesis [48]. In our previous work, we detected a direct correlation between miR-138.5p, with pallidum and amygdala size [15]. However, in this study, miR-138.5p was negatively associated with NEDA-3. These contradictory results make us question the real value of this microRNA as a biomarker, but we conclude that it would more probably hurt neuroprotection, regarding this latest data (follow-up design with clinical endpoints more meaningful than MRI metrics in a cross-sectional study), and the bigger evidence in this direction. 

Finally, we did not find any relationship with any microRNAs at 5 years of follow-up. This lack of association could be explained by methodological reasons because of a greater heterogeneity after that time of evolution. 

### Limitations Section

We are aware of some limitations and weaknesses of our study. First of all, the study was conducted on a small number of participants. This was an exploratory study, and we think that we could overcome this limitation thanks to the great homogeneity of our study population and with a preselection of the target miRNAs through a systems biology approach that enabled us to reduce the number of miRNAs to be studied in such a small sample. This methodology was proven effective and adequate in a previous study [15]. In the same way, we did not use a control group. This data would have added valuable information, but we think it was not completely necessary for the objective of our research, as we were not interested in analyzing the differences in miRNAs profiles between MS and healthy subjects or regarding different treatments, which have been previously studied in other articles [5,6,7,49,50,51,52,53,54], but in investigating the utility of a set of miRNAs as predictors of the clinical evolution of MS itself. We chose GA for this reason, to get a homogeneous group representative of an early phase of MS (which would reduce some variability), and because the cleaner metabolism of GA compared to other treatments would minimize the changes in miRNAs expression caused by other factors unconnected to the main mode of action of the drug, as it has been previously suggested [55,56]. We also understand that there are some limitations regarding the statistics applied in the study, mainly regarding multiple tests without correction for multiple comparisons. Non-parametric tests are normally more robust. Due to the exploratory nature of this study, we decided that Bonferroni’s corrections could be very stringent. We are aware that without this correction, we are risking some type I errors, but with very hard correction criteria, we could also be increasing type II errors and losing some less powerful statistics but clinically interesting correlations. We think that the results with *p* < 0.05 deserve to be exposed and discussed, mainly given the multiple and coherent results obtained, especially with mir-146a.5p and with mir-9.5p. Finally, it will be necessary to replicate these results in larger and independent cohorts to confirm the effects of these microRNAs in MS and validate their applicability to monitor the progression of the disease and their utility as serum biomarkers.

## 5. Conclusions

This article reinforces the implication of microRNAs in the pathogenesis and evolution of multiple sclerosis. It would be of great interest to further investigate the role of these microRNA in this disease to elucidate their possible utility as prognostic biomarkers, predictors of response to disease-modifying treatments, and even as therapeutic agents.

## Figures and Tables

**Table 1:** List of microRNAs included in the study. Clinical and radiological associations found in previous research.

microRNas	Clinical Association	MRI Volume
9.5p	EDSS	Thalamus
126.3p	SDMT	-
138.5p	-	Pallidum and amygdala
146a.5p	EDSS and SDMT	-
200c.3p	-	Cerebellum and pallidum
223.3p	-	Caudate

EDSS: Expanded Disability Status Scale. SDMT: Symbol Digit Modalities Test.

**Table 2:** Epidemiological data.

Sex N (F:M)	Age at MS Onset (Years) Md (ICR)	Age at GA Onset (Years) Md (ICR)	Time with GA at Study Onset (Years) Md (ICR)	Basal EDSS Mean (±SD)
18:8	31.9 (25.1–41.9)	32.8 (26.6–44.9)	4 (2.1–6.4)	1.4 (1.7)

N: number. F: Female. M: male. Md: Median. ICR: Interquartile range. SD: Standard Deviation. MS: Multiple Sclerosis. GA: Glatiramer acetate. EDSS: Expanded Disability Status Scale.

**Table 3:** Population distribution at 2 and 5 years.

Clinical Data	2 Years (n = 25)	5 Years (n = 24)
NEDA-3	70.8% (n = 17/24)	56.5% (n = 13/23)
Relapse	8% (n = 2)	13% (n = 3)
6-month CDP	12% (n = 3)	17.4% (n = 4)
MRI activity	8% (n = 2)	13% (n = 3)
Treatment GA	60% (n = 15)	20.8% (n = 5)
GA failure	28% (n = 7)	42.3% (n = 11)
GA change (not failure)	12% (n = 3)	30.8% (n = 8)

NEDA-3: No Evidence of Disease Activity. n = number of patients. CDP: Confirmed Disease Progression. GA: Glatiramer acetate.

**Table 4:** Associations between miRNAs and EDSS progression at 2 and 5 years (backstep multivariate regression analysis).

**miRNA**	**EDSS 2 Years**	
	**β (CI 95%)**	***p***
9.5p	0.23 (0.04–0.46)	* 0.047
	Excluded variables (β; *p*)	
126.3p	−1.94; 0.554	
138.5p	2.45; 0.396	
146a.5p	−3.21; 0.262	
200c.3p	−1.03; 0.764	
223.3p	1.9; 0.583	
**miRNA**	**EDSS 5 Years**	
	Excluded variables (β; *p*)	
9.5p	1.41; 0.697	
126.3p	0.36; 0.305	
138.5p	−2.92; 0.413	
146a.5p	0.41; 0.234	
200c.3p	0.36; 0.305	
223.3p	−2.95; 0.408	

Expanded Disability Status Scale. CI: Confidence Interval. * statistically significant *p* value

**Table 5:** Summary of the results of the microRNAs included in the study.

miRNA	Clinical Evolution (2 Years)			
	EDSS	Relapse/MRI Activity	CDP	NEDA-3
	(Multivariate Regression Analysis)	(U Mann–Whitney Test)		
9.5p	+	-	-	-
126.3p	-	-	+	-
138.5p	-	-	-	+
146a.5p	-	-	+	-
200c.3p	-	-	-	-
223.3p	-	-	-	-
	Clinical evolution (5 years)			
No miRNAs associated with any clinical/radiological variable				

EDSS: Expanded Disability Status Scale. CDP: Confirmed Disability Progression. NEDA-3: No Evidence of Disease Activity.

## Data Availability

The data that support the findings of this study are available on request from the corresponding author.

## Supplementary Materials

The following supporting information can be downloaded at: https://www.mdpi.com/article/10.3390/biomedicines11102760/s1, Table S1: Correlations between miRNAs and relapse/MRI activity, CDP and NEDA-3 at 2 and 5 years (U Mann–Whitney).
