# Supplementary material for: MicroRNAs Associated with Disability Progression and Clinical Activity in Multiple Sclerosis Patients Treated with Glatiramer Acetate
Source: Biomedicines. 2023 Oct 12;11(10):2760. doi: 10.3390/biomedicines11102760 (PMC10604830; doi:10.3390/biomedicines11102760)
Supplement: Supplementary file 1 [file biomedicines-11-02760-s001.zip › biomedicines-2651639-supplementary.pdf]

Table S1. Correlations between miRNAs and relapse/MRI activity, CDP, and NEDA-3 at 2 and 5 years (U Mann-Whitney).

**miRNA**	**EDSS 2 years**		
	Relapse/MRI activity (p)	CDP	NEDA-3
9.5p	0.885	0.584	0.673
126.3p	0.128	*0.05	0.924
138.5p	0.059	0.223	*0.033
146a.5p	0.303	*0.044	0.634
200c.3p	0.823	0.655	0.883
223.3p	0.37	0.368	0.24

**miRNA**	**EDSS 5 years**		
	Relapse/MRI activity	CDP	NEDA-3
9.5p	0.355	0.926	0.491
126.3p	0.357	0.428	0.828
138.5p	0.425	0.275	0.257
146a.5p	0.759	0.141	0.306
200c.3p	0.916	0.903	0.99
223.3p	0.598	0.484	0.804

EDSS: Expanded Disability Status Scale. CDP: Confirmed Disability Progression. NEDA-3: No Evidence of Disease Activity. * statistically significant *p* value.
